# Toward development of optimum specimen designs and modeling of in-plane uniaxial compression testing of aluminum alloy 2024 and AISI 1008 steel sheet material

**DOI:** 10.1088/1742-6596/1063/1/012068

**Published:** 2018

**Authors:** D K Banerjee, C A Calhoun, M A Iadicola, W E Luecke, T J Foecke

**Affiliations:** 1 Material Measurement Laboratory, NIST, Gaithersburg, Maryland, USA

## Abstract

Tension-compression testing is commonly conducted to understand and predict springback during a stamping process. However, large strains are generally difficult to achieve during the in-plane compression portion of the test. Proper specimen design and control of frictional forces are necessary for obtaining large strains. This paper describes extensive finite element analyses (FEA) and optimization studies (Phase 1) that were conducted to calibrate the model test assembly for three different buckling modes obtained in uniaxial compression tests of aluminum alloy 2024 and American Iron and Steel Institute (AISI) 1008 steel specimens. In addition to obtaining these three buckling modes correctly, calibrated FEA model predicted forces matched measured forces reasonably well. Also, a good agreement between computed and measured stress-strain data was demonstrated for one compression experiment. In the Phase 2 optimization study, optimum specimen geometries will be developed by using these verified, optimum FEA model test assemblies in three types of compression buckling experiments.

## 1. Introduction

Springback often occurs in sheet metal forming and its prediction poses one of the more complicated modeling challenges. Springback results due to through thickness residual stresses that develop during the complex loading associated with the stamping process, and can cause significant spatial distortions in stamped parts. Tension-compression testing, in conjunction with numerical modeling, is commonly conducted to help understand and predict springback during a stamping process. Large strain is generally difficult to achieve during the in-plane compression portion of the test, since sheet material specimens can exhibit multiple buckling modes. Therefore, a careful design of the specimen geometry is needed for deformation of specimens to large strains during combined tension and compression tests, even when antibuckling guides are used. Compression testing of sheet materials is typically performed using dog bone specimens having length to width ratio of 3[1]. The efficacy of compression tests depends on the ratio of gauge length to thickness of the specimen. Researchers have obtained in-plane compression strains ranging from 0.01 to 0.15 by varying the gauge length/thickness ratios from 16 to 2 [2,3]. Specimen design is crucial since it influences the largest unsupported length that can be used to obtain the maximum plastic strain in the specimen. A hybrid approach, employing both small specimen size and side plate supports applying lateral forces, can help improve the maximum strain that can be achieved, but the approach is still vulnerable as buckling can result both in the supported and the unsupported regions. The state of stress is strongly influenced by the lateral force and the coefficient of friction at the interface between the specimen/side plates and the specimen/tab region. The study of plastic flow behavior is affected by the presence of friction. Additionally, any variation in frictional forces can influence the uniformity of the stress field. Several modes of buckling have been reported in the literature [4].

**Figure 1.**
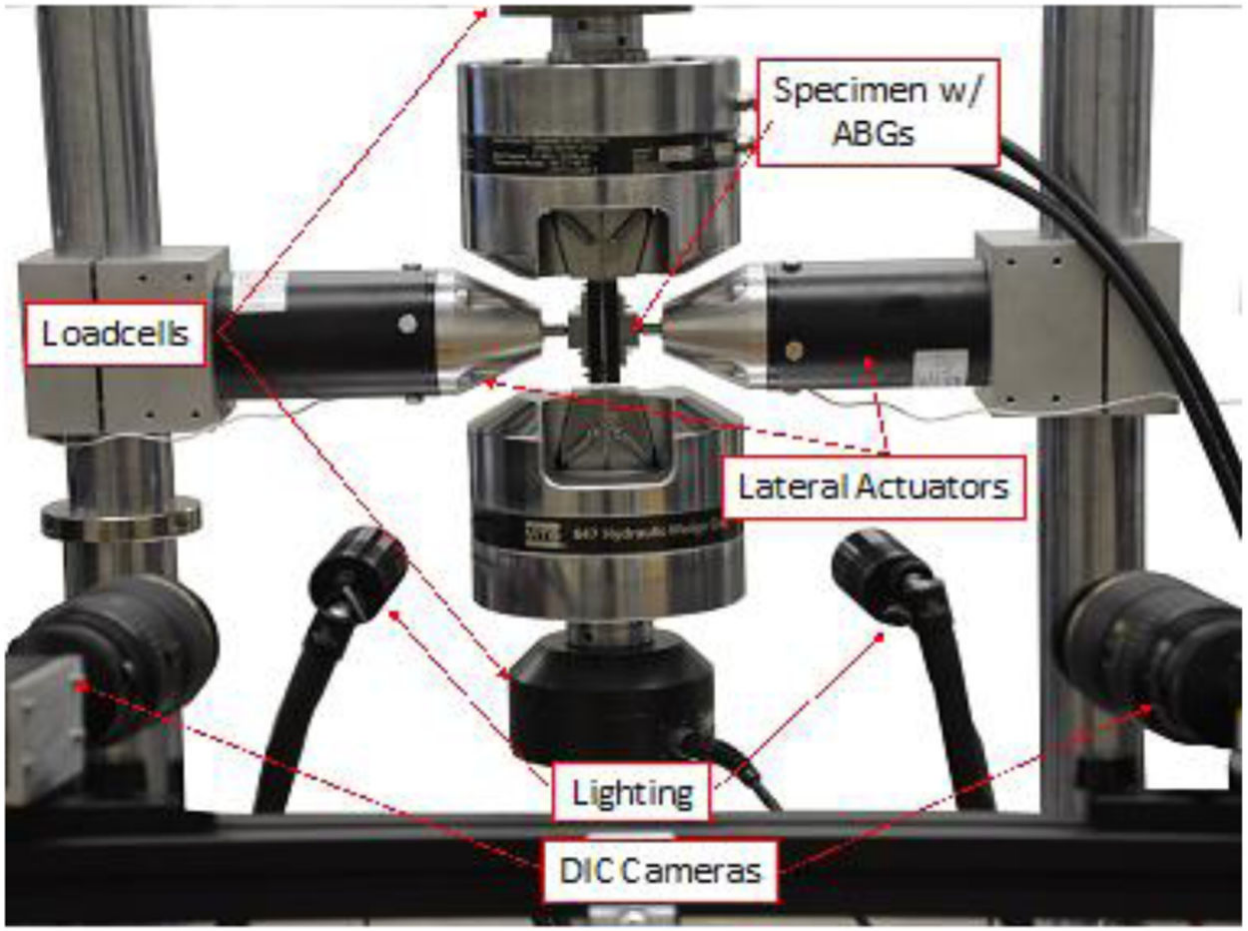
Setup with DIC, ABGs, and two load cells.

An extensive finite element analysis (FEA) and optimization study are planned to identify and optimize aluminum alloy 2024 and AISI 1008 steel compressive test specimen geometries. In this work (Phase 1), the model parameters are verified using a combination of simulated and measured data obtained with the experimental set up shown in figure 1. The paper is organized as follows. First an overview of mechanical tests is provided followed by a discussion on modeling of tests using FEA. Finally, an insight into Phase 1 optimization studies is provided for the three types of buckling modes (two described in [4] and one additional mode). The Phase 1 optimization goal is to obtain the three buckling modes and determine unknown modeling parameters (e.g., friction coefficients). Phase 2 is planned to use this verified model to optimize the specimen geometry.

## 2. Overview of compression tests conducted

**Figure 2a.**
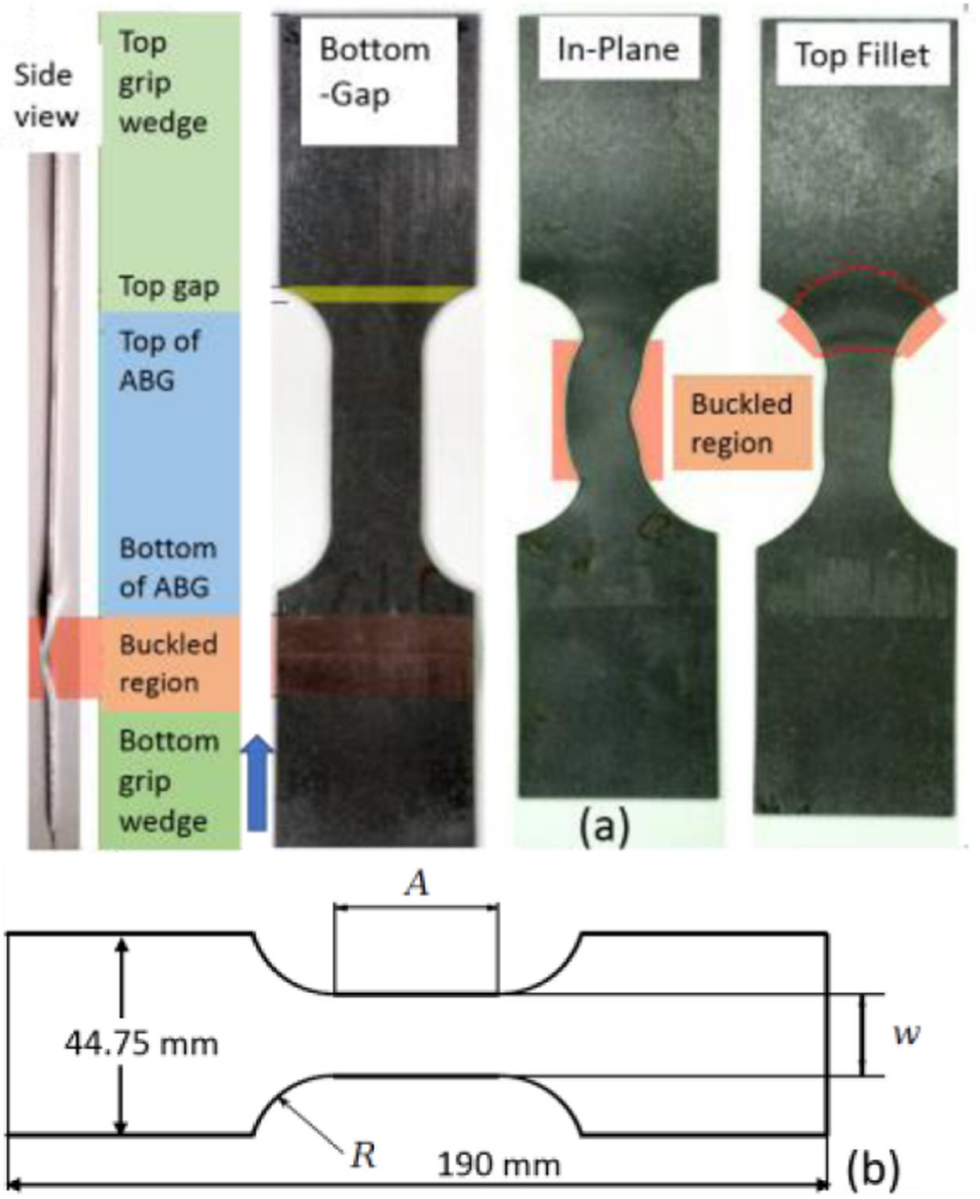
Buckling modes in compression tests; 2b. Schematic specimen sketch.

Uniaxial compression tests were conducted using a new experimental set up (an improvement over the one described in [8]) that uses digital image correlation (DIC) for displacement and strain measurement. Solid, flat plates (anti-buckling guide, ABG) along with a lateral force were used for out-of-plane buckling restraint. An initial special specimen design was chosen based on a parametric study with the goal to minimize buckling outside of the restrained region. Applied constant lateral plate force allows for more reliable biaxial and friction corrections than in tests where the support is provided by plates fixed in lateral position in opposition to the changing thickness of the specimen. For a short column like region (e.g., the unsupported region of the specimen) failure is caused by plastic yielding as opposed to buckling. Depending on the variations in specimen geometry parameters, types of material considered, and magnitude of the lateral forces, three different buckling modes were observed: a) bottom gap or *L*-buckling [4], b) in-plane buckling or *W*-buckling [4], and top fillet buckling (not seen in [4]). Uniaxial compression tests were conducted using different specimen designs (table 1 and figure 2(b)) in order to obtain the three different buckling modes using the setup shown in figure 1. This employs a modified servo-hydraulic test frame, where strains were measured using a DIC system that uses two 9.1-megapixel cameras. Load cells above and below the hydraulic grips permit the measurement of the frictional forces applied to the specimen by the ABGs. Two pneumatic lateral actuators attached to the vertical supports of the load frame impart a constant lateral force to the specimen. The pressure in the cylinders was held constant throughout the tests. Ball and socket joints between the ABGs and load cells ensure that the faces of ABG plates and specimen faces remain parallel. Teflon film and petroleum jelly were used on the specimen/ABG interfaces for lubrication and minimization of friction. The DIC acquisition is conducted on the exposed side of the specimen (thickness direction) that was not obscured by the ABGs. A virtual extensometer of about 25 mm length is selected in the middle of the gauge region. Strains are computed by the DIC software by knowing the initial 3D coordinates and monitoring the displacements and extension of this virtual extensometer. Three different buckling modes were considered: bottom gap buckling or *L*-buckling, in-plane or *W*-buckling, and top fillet buckling using different specimen geometries. Table 1 lists key parameters defining the geometry of each of these specimens. Note that two different specimen geometries were used for in-plane buckling tests. Figure 2(a) shows images of buckled specimens for the three buckling modes. Figure 2(b) shows a schematic of the specimen design. For the bottom gap buckling, aluminum alloy 2024 was used. AISI 1008 steel specimens were used for the two other buckling mode specimens. A combination of the specimen geometry, material properties, the axial loading, lubrication, and the lateral forces applied through the ABGs will ultimately determine the type of buckling. All the test specimens were cut from sheets by waterjet. Sheet thickness was 1.14 mm for all tests except for the Al 2024 specimens, which were 1 mm thick. All tests were conducted under displacement control. Strain rates ranged from 4x10^−4^ s^−1^ to 6.9x10^−4^s^−1^. The top of the specimens was fixed and compressive vertical displacement was applied to the bottom grip by the hydraulic actuator. Force-displacement data for all tests were recorded and compared with FEA simulations for calibration of the model and parameter optimization.

### 2.1 Bottom gap buckling (L-buckling)

The bottom gap buckling study was conducted using six aluminum alloy 2024 specimens summarized in table 2. The unsupported length at the top of the specimen was always 3.45 mm. Uniaxial compression experiments were run with different initial bottom gap spacing. Table 2 also lists mean lateral forces. The buckling can be compared with the well-known Euler buckling [5]. The constitutive material data needed for the FEA study were generated by conducting uniaxial tensile stress on the S160609-ERR-100 specimen (see table 2).

### 2.2 In-plane (W-buckling)

The in-plane buckling experiments were conducted using AISI 1008 steel specimens. All tests were conducted with 3.6 kN lateral force applied to specimens by the ABGs. As positioned in the test frame, 3.45 mm at the top and 19.8 mm at the bottom of the specimen length were unsupported. Five tests were conducted with the In-plane1 specimen geometry (table 1), with net uniaxial displacements of 7 mm (one test), 9 mm (two tests), and 18 mm (two tests). All specimens except the one with 7 mm net displacement buckled. A separate in-plane compression test to a 4.8 mm displacement was conducted with the In-plane2 specimen to demonstrate the uniformity of deformation achieved in the gauge section. Another In-plane2 specimen was tested in tension to generate the constitutive material data.

### 2.3 Top fillet buckling

Three identical tests were conducted for the top fillet buckling tests. The unsupported specimen length at the top and bottom were the same as they were for the in-plane buckling tests. The mean lateral forces applied to the specimens by the ABGs were half of those that were applied for the case of bottom gap and in-plane buckling, i.e., 1.8 kN. Buckling in the top fillet buckling specimen always occurred on the top of the specimen (opposite the actuator). The interaction of dynamic and static friction may be the cause.

## 3. FEA and Phase 1 optimization described

**Figure 3.**
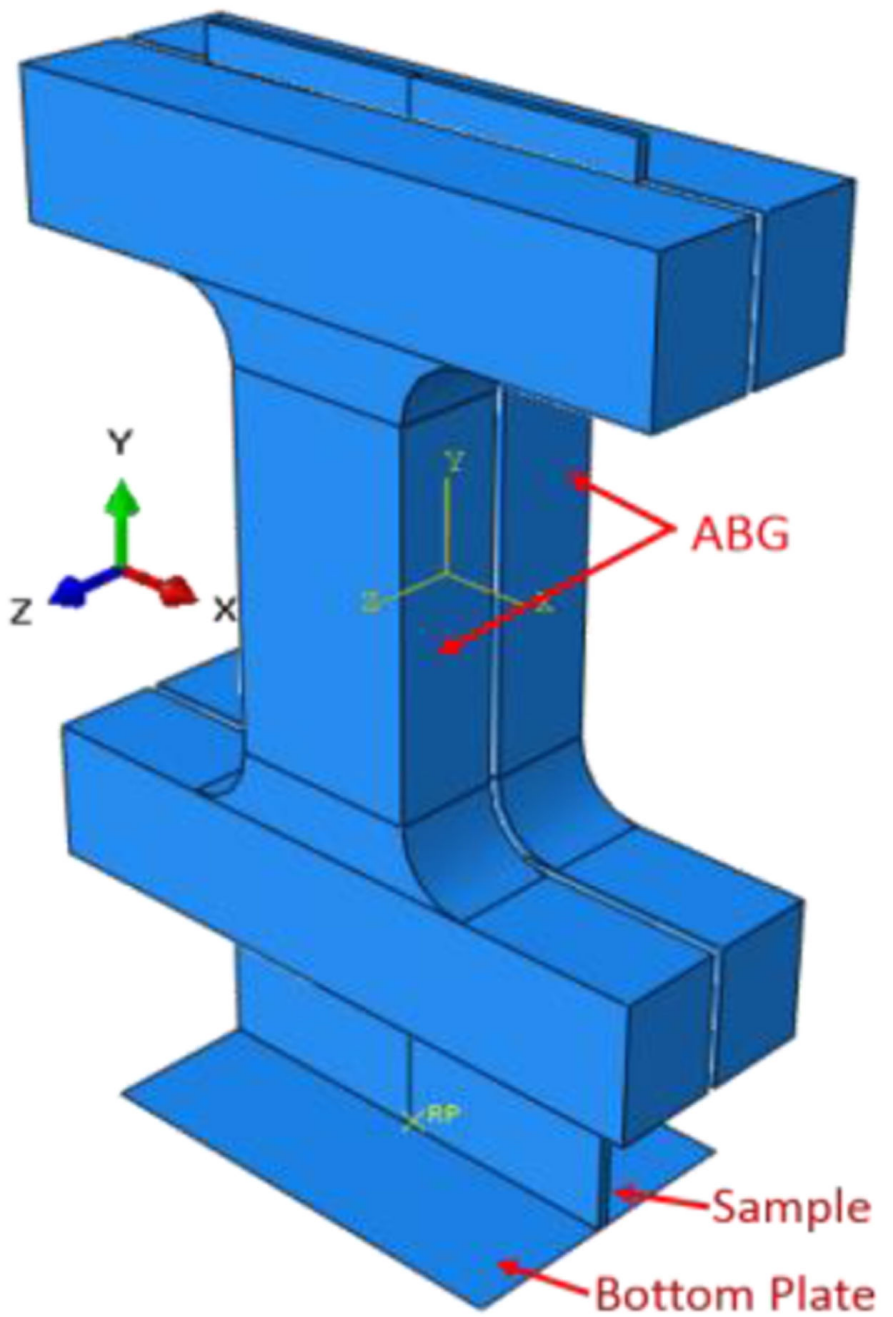
FEA model assembly with ABGs and bottom plate.

**Figure 4.**
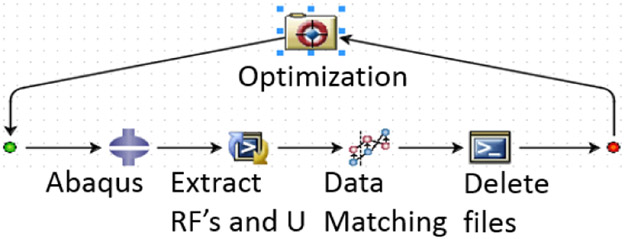
Isight optimization process flow.

After the trial geometry was determined, FEA simulation is used to obtain an optimized specimen test configuration. Figure 3 shows the FEA test assembly with ABGs and a bottom plate to approximate the imperfect boundary condition at the bottom grip face. In the Phase 1 optimization, the FEA model parameter values such as the coefficient of friction between the ABGs and the specimen and that between the specimen bottom and the bottom plate and a small numerical force imperfection (explained later) were optimized by minimizing the differences between measured and FEA obtained force-displacement data in Isight software [6]. The Isight process flow includes a procedure where initial model parameters are improved by repeated runs of the FEA model in Abaqus software [7] (see figure 4). The FEA model of the specimen was constructed in Abaqus using appropriate specimen dimensions and test assembly configurations for each type of buckling study. Linear hexahedral, reduced integration elements, C3D8R, were used along with mapped meshing. Material properties (except constitutive material behavior data) are given in table 3. Isotropic hardening was used in all FEA models as very little kinetic hardening is seen for 1008 steel specimens [8]. Isotropic hardening was also assumed for Al 2024 specimens. Both the specimen and ABGs were classified as deformable bodies. Material properties for both specimen and ABGs were defined. The bottom plate (by which the compressive displacement is applied) was defined as an analytical rigid body. Surface-to-surface contacts were created between specimen surfaces and each of the two ABGs and initial Coulomb friction (constant value) was chosen for interaction between specimen and ABGs. Additional surface-to-surface contacts were created between the specimen bottom and the bottom plate with a different value of initial Coulomb friction for interaction. The kinematic contact method with finite sliding was used as the mechanical constraint formulation in Abaqus/Explicit [7]. The Phase 1 optimization process flow is shown in figure 4. The design variables are two friction coefficients at the specimen/ABG and the specimen bottom/the bottom plate interfaces, and a very small numerical force imperfection that was applied at an appropriate location (which varies for the three types of buckling studies) to introduce buckling instability (see below). The objective function is the minimization of the sum of square of differences between the measured and computed forces at the top of the specimen for the entire displacement history $\left(\Delta F_{sum}^{2}\right)$. The NLPQLP [5] optimization algorithm used is a special implementation of a sequential quadratic programming (SQP) method. In this method, a quadratic programming subproblem is formulated and solved by conducting a quadratic approximation of the Lagrangian function and a linearization of constraints. It is a gradient based method and is well suited for problems with continuous design spaces. For each optimization run with a given set of values for the design parameters, Abaqus is run and the force outputs at specimen top and displacements are extracted in the module “Extract RF’s and U”. A Python script is executed by this module to extract these data from Abaqus output database (ODB) file. These computed data (reaction force vs. displacement) are then compared with measured data in the “Data Matching” module. The ODB files, which are no longer needed, are then deleted in the “Delete Files” module. This sequence of operations is continued for each run. Isight determines the optimal model parameters within the explored space. These values of model parameters are then used for all runs for the study of that type of buckling. Note that U1, U2, U3 are FEA displacements in X, Y, and Z directions.

### 3.1 Bottom buckling FEA and Phase 1 optimization study

**Figure 5.**
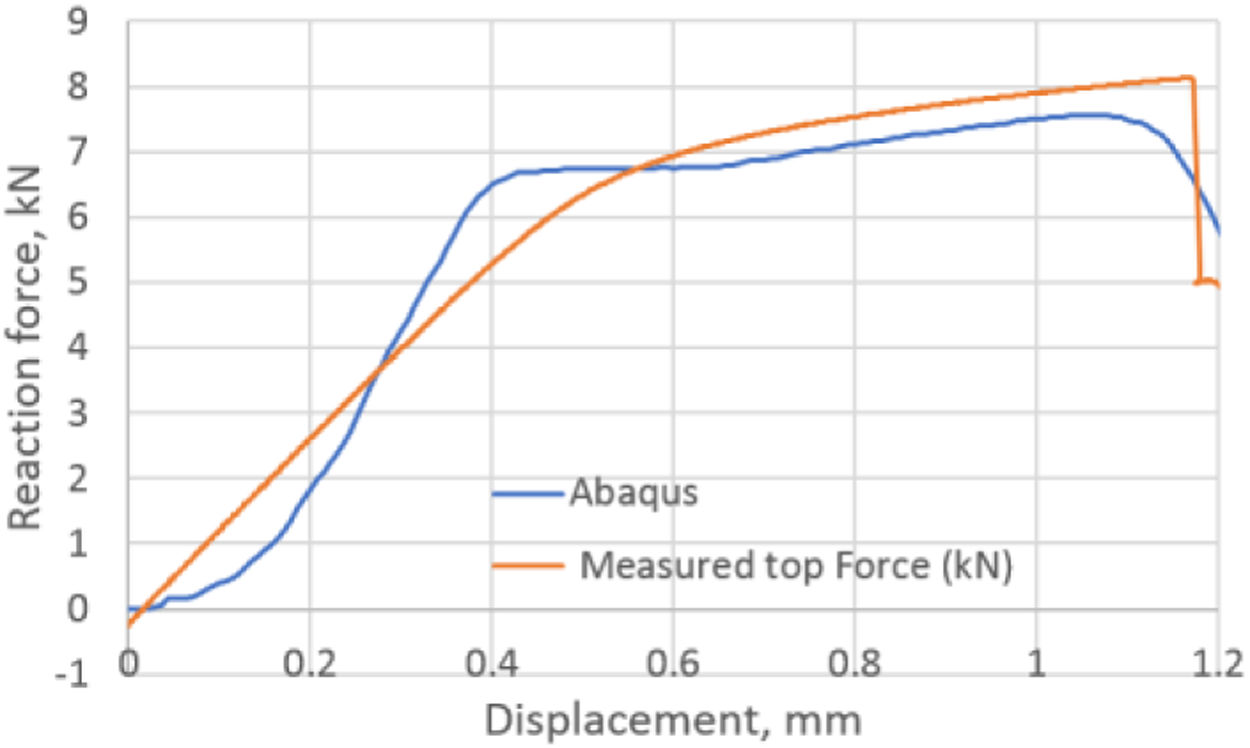
Measured and optimum FEA reaction forces at top vs. displacement.

**Figure 6.**
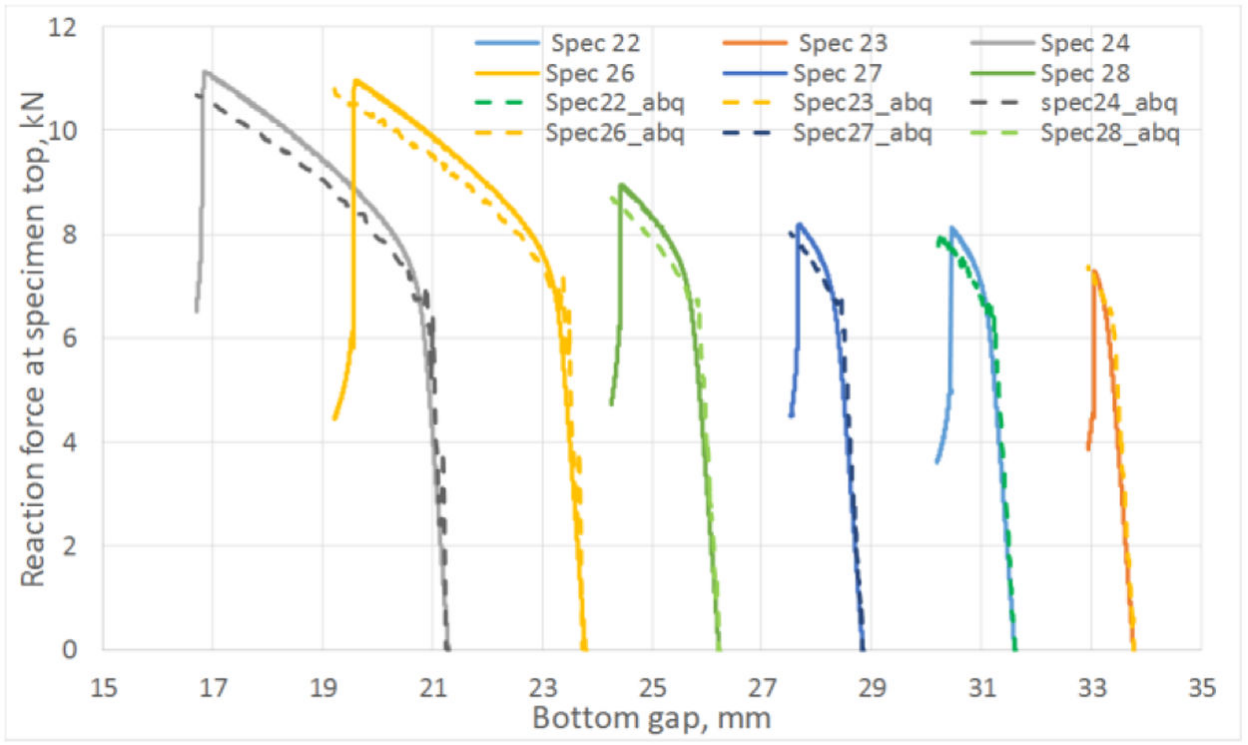
Measured and FEA reaction forces (after Phase 1 optimization) at specimen top vs. bottom gap for all tests in table 2.

**Figure 7.**
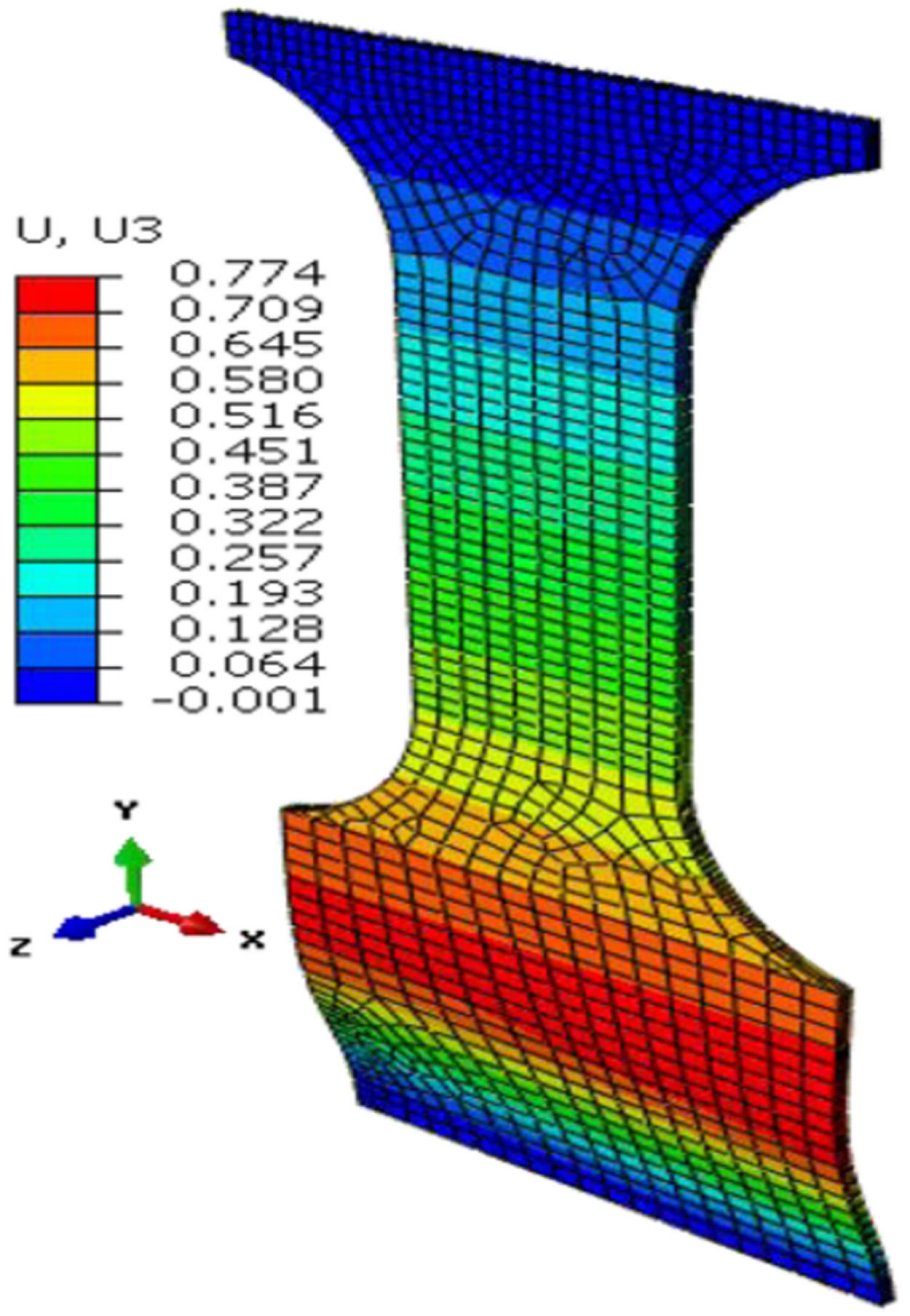
Optimum FEA predicted shape and U3 displacement for specimen 22 (table 2).

For the bottom buckling experiments, the Phase 1 optimization was conducted with specimen S160418-DJP-022 (see table 2). For this analysis, only two design variables were chosen, i.e., the friction coefficient at the specimen/ABG and specimen/bottom plate interfaces (which are the same). No force imperfection for inducing instability was applied. The optimum value of the friction coefficient was found to be 0.0719. Subsequently, this value was used for all the analyses for tests listed in table 2 and shown in figure 6. A comparison between measured and computed reaction forces at the top of the specimen is shown in figure 5 for the optimum run. Also, a comparison between measured and computed reaction forces at the specimen top as a function of bottom gap for all the tests is shown in figure 6. Figure 7 shows a typical buckling result (not to scale).

### 3.2 In-plane and top fillet buckling FEA and Phase 1 optimization study

**Figure 8.**
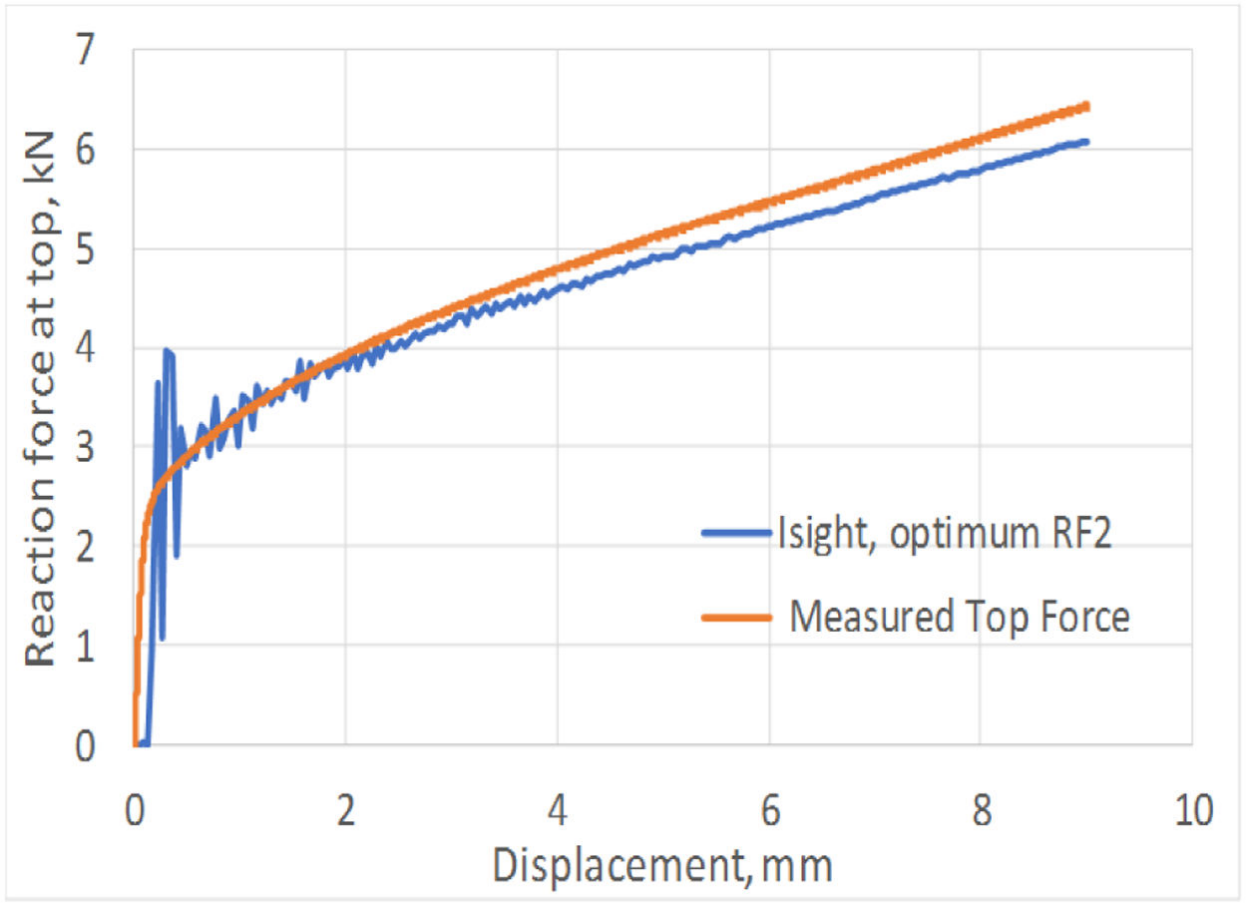
Measured and optimum FEA reaction forces at top vs. displacement for In-plane 1 buckling specimen.

**Figure 9.**
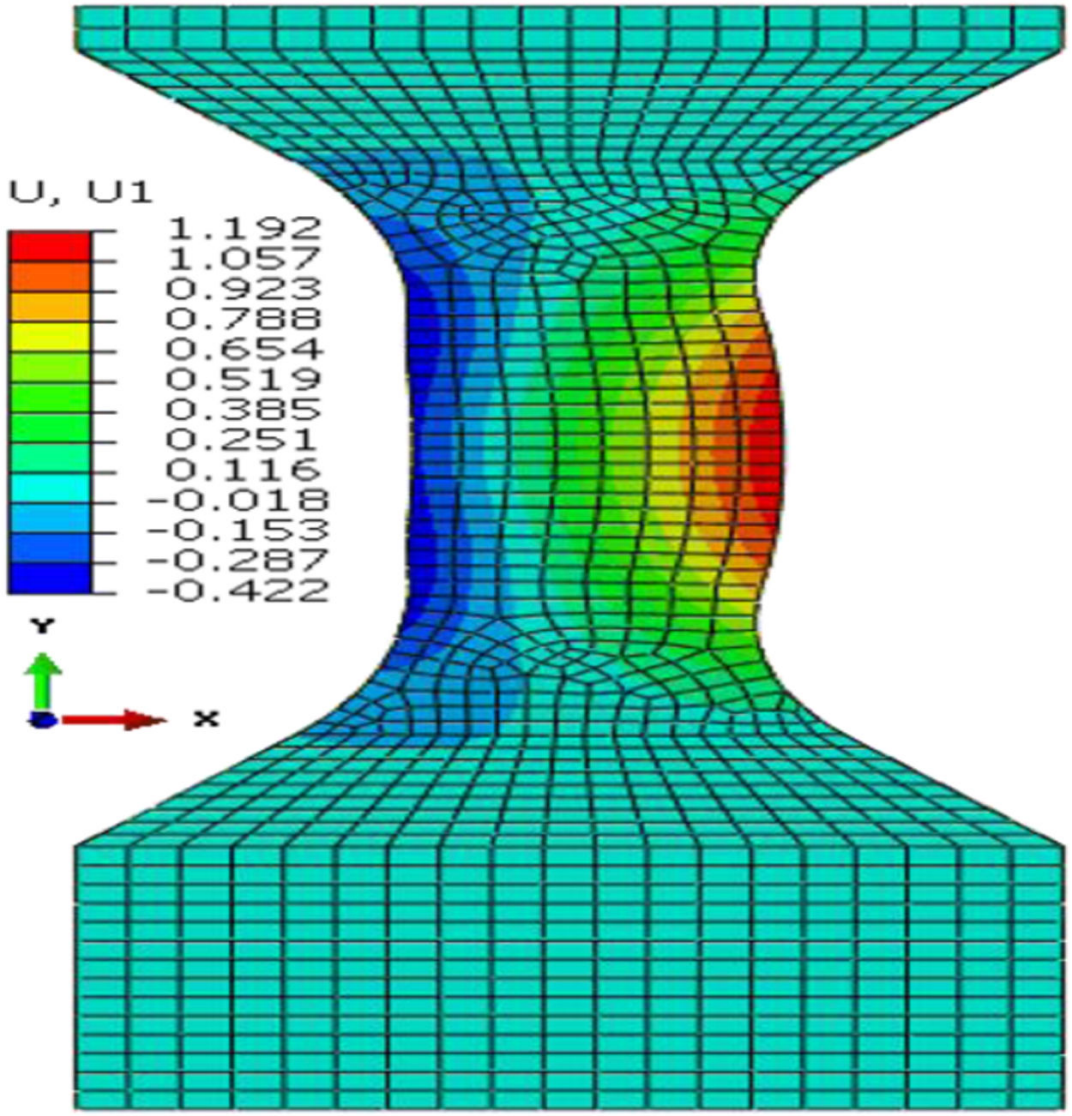
FEA predicted shape and U1 for In-plane 1 specimen (optimum run).

**Figure 10.**
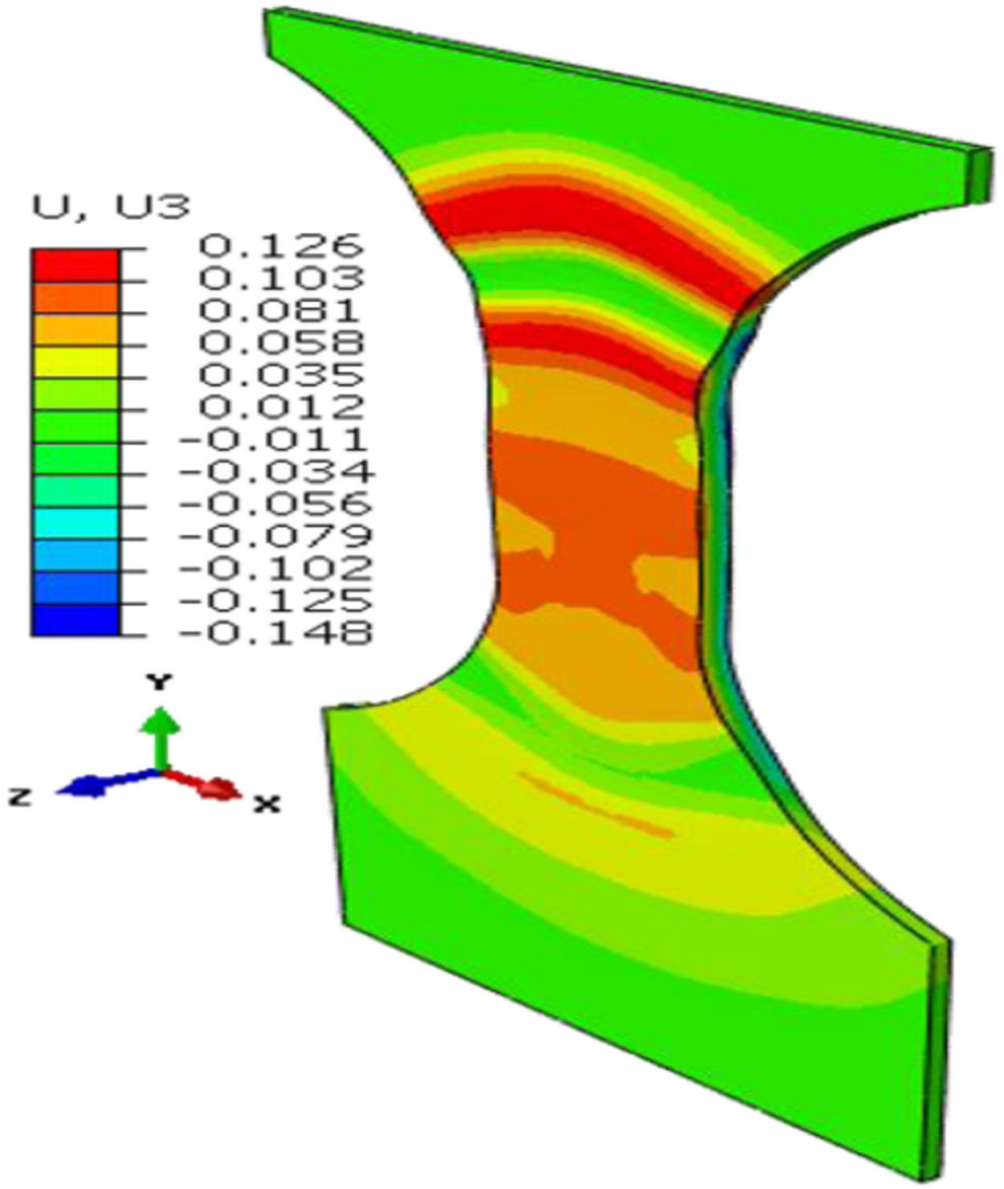
FEA predicted shape and U3 for top fillet buckling specimen (optimum run).

**Figure 11.**
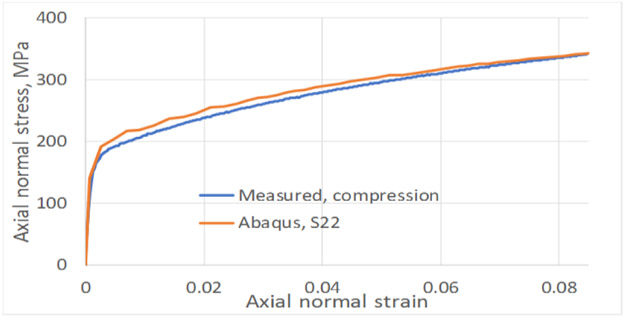
Measured and optimum FEA axial normal stress vs. strain for In-plane 2 buckling specimen (table 1).

The FEA assembly models for both of these studies were similar as the top and bottom gap (unsupported lengths) were identical (e.g., 3.45 mm and 19.8 mm respectively). The differences are in specimen geometric parameters (figure 2) and the lateral forces applied through ABGs. The lateral force for the top-fillet buckling tests was 1.8 kN, half of that in in-plane test. For the in-plane buckling, a small numerical force imperfection (L_instab_) was applied to induce instability in the X-direction at a node located at mid-point of the gauge area (along the plane of the specimen). For the top-fillet buckling, that force imperfection was applied in the out-of-plane Z-direction at one node on the middle of the top-fillet region. For each study, the FEA constitutive model calibration strategy was similar. Stress-strain data used for the FEA model for both problems came from a uniaxial tensile test using the specimen design for the In-plane2 geometry in table 1. In the Phase 1 optimization study, friction coefficients (at ABG, fric1 and bottom, fric2) and the force imperfection for inducing instability (L_instab_) were the model parameters optimized. The objective function was minimization of $\Delta F_{sum}^{2}$. For the in-plane buckling the optimum design parameters were fric1=0.011, fric2=0.69, L_instab_ =5.462 N. Figure 8 shows compares the reaction forces at specimen top for the experiment to the Phase 1 optimum FEA run. A typical plot showing the specimen deformed shape is shown in figure 9 (not to scale). For top-fillet buckling specimen, the match of reaction forces was similar (not shown). Figure 10 shows the final shape (not to scale) of the optimum FEA run with out of plane displacement contour plot. For the top fillet buckling, the Phase 1 optimum model parameters were fric1=0.010, fric2=0.494, L_instab_ =2.26 N. Figure 11 shows the stress-strain data (measured vs. FEA) for the case of the In-plane2 specimen (table 1) using the Phase 1 optimum model parameters for the in-plane optimum run as mentioned above. For the FEA result, the axial normal stress and strain data were collected by taking average of all nodal values along a 25 mm long virtual extensometer positioned in the middle of the specimen gauge length. The FEA results show a slight over-prediction of stress.

## 4. Discussion and future study

**Figure 12.**
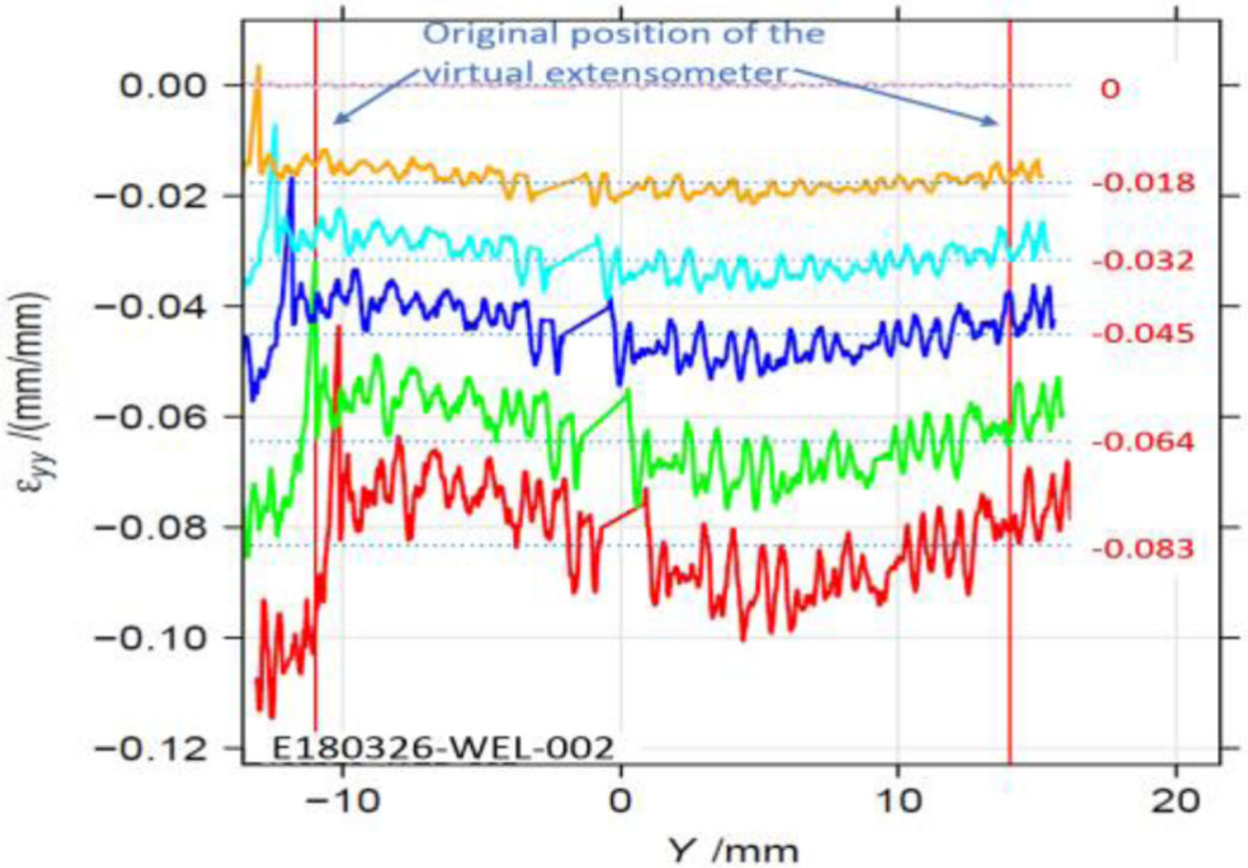
DIC *ε*yy along reduced parallel length of the In-plane2 specimen (table 1).

For the in-plane compression testing using the model setup described here, uncertainties in the stress measurements and nonuniformity in strain measurement are due to frictional forces at specimen/ABG interface. Hence, frictional forces must be minimized. Note that the use of two load cells allows for a measurement of the average frictional forces on the entire interface, not just the gauge area. For the bottom buckling tests, very little plastic strain was achieved except in specimen 24 and 26. This is due to final net displacement prior to buckling of less than 2 mm. Figure 6 shows a reasonable match of reaction forces for all six tests, except toward the end of the tests. Assumption of constant values of friction coefficient may be the reason. The same is true for top fillet and in-plane tests (figure 8). The reason behind oscillations in FEA reaction forces seen for in-plane (figure 8) tests is not clear. This is possibly due to how Abaqus [7] calculates friction on a discrete surface. The forces tend to jump for dissimilar meshes belonging to each of the two contacting surfaces, when nodes slide past each other. Desired buckling shapes were obtained in optimized FEA tests (figure 7, 9, 10). Agreement in stress-strain data is very good (figure 11). The error in estimation of initial yield strength (at zero plastic strain) in material model used in FEA may be the cause of the slight discrepancy. The assumption of isotropic hardening could also contribute to this error. Strain uniformity is desired in the gauge length of the virtual extensometer. This indeed was seen in test (figure 12) and optimum FEA results (not shown). Figure 12 shows local *ε*yy at several true strain levels during compression, where horizontal lines denote the average value of the local true strain computed with gauge length of 25 mm. The optimum values of friction coefficients obtained at specimen/ABG interface for both top fillet and in-plane buckling tests were similar to those reported in ref [8]. Specimen geometry and boundary conditions (ABG friction and lateral forces) determine which buckling mode is likely to occur. Fillet geometry and lateral forces seem to have a strong influence on top fillet buckling, opposite to the moving actuator. In Phase 2 of the optimization study, optimum specimen design parameters (figure 2b) will be obtained that maximize plastic strain in gauge region before the onset of buckling. Identification of this onset is mathematically difficult. But one possible approach is to identify the point at which the positional strain rate gradients (*dε/dx* etc.) in specimen areas of interest show an abrupt and significant change.

## Figures and Tables

**Table 1.** Specimen geometric parameters.

Specimen	R (mm)	A (mm)	w (mm)
Bottom gap	16	40	20
Top fillet	26	24	12
In-plane 1	19	36	12
In-plane 2	19	36	18

**Table 2.** Bottom gap tests with lateral forces and displacements.

Specimen	Bottom gap (mm)	Displace ment (mm)	Side Force (kN)	Tension/ compr ession
S160418-DJP-022	31.63	1.43	3.576	C
S160418-DJP-023	33.78	0.84	0.858	C
S160418-DJP-024	21.31	4.6	3.586	C
S160418-DJP-026	23.8	4.58	3.583	C
S160418-DJP-027	28.85	1.32	3.57	C
S160418-DJP-028	26.24	1.97	3.56	C
S160609-ERR-100	-	12.44	-	T

**Table 3.** Material properties used.

Material	Young's Modulus GPa	Yield Strength MPa	Poisson's ratio	Density kg/m^3^
Al Alloy 2024	73.1	324	0.3	2780
Steel (ABG)	210	236.4	0.3	7890
AISI 1008 (Steel)	192	195	0.3	7872
